# Three-dimensional greyscale transrectal ultrasound-guidance and biopsy core preembedding for detection of prostate cancer: Dutch clinical cohort study

**DOI:** 10.1186/s12894-019-0455-7

**Published:** 2019-04-16

**Authors:** Anouk Anna Maria Arnoldus van der Aa, Christophe Koenraad Mannaerts, Maudy C. W. Gayet, Johannes Cornelis van der Linden, Barthold Philip Schrier, J. P. Michiel Sedelaar, Massimo Mischi, Harrie P. Beerlage, Hessel Wijkstra

**Affiliations:** 1 Department of Urology, Jeroen Bosch Hospital, Post office box 90153, ‘s-Hertogenbosch, 5200 ME The Netherlands; 2 Department of Pathology, Jeroen Bosch Hospital, ‘s-Hertogenbosch, The Netherlands; 3 Department of Urology, Radboud University Medical Centre, Nijmegen, The Netherlands; 4 Electrical Engineering, Eindhoven University of Technology, Eindhoven, The Netherlands; 5 Department of Urology, AMC University Hospital, Amsterdam, The Netherlands

**Keywords:** Prostatic neoplasms, Biopsy, Three-dimensional imaging, Tissue embedding, Diagnosis

## Abstract

**Background:**

To overcome the limitations regarding two dimensional (2D) greyscale (GS) transrectal ultrasound (TRUS)-guided biopsy in prostate cancer (PCa) detection and tissue packaging in biopsy processing, there is an ongoing focus on new imaging and pathology techniques. A three-dimensional (3D) model of the prostate with biopsy needle guidance can be generate by the Navigo™ workstation (UC-care, Israel). The SmartBX™ system (UC-care, Israel) provides a prostate biopsy core preembedding method. The aim of this study was to compare cancer detection rates between the 3D TRUS-guidance and preembedding method with conventional 2D GS TRUS-guidance among patients undergoing prostate biopsies.

**Methods:**

We retrospectively analyzed the records of all patients who underwent prostate biopsies for PCa detection at our institution from 2007 to 2016. The cohort was divided into a 2D GS TRUS-guidance cohort (from 2007 to 2013, *n* = 1149) and a 3D GS TRUS-guidance with preembedding cohort (from 2013 to 2016, *n* = 469). Effect of 3D GS TRUS-guidance with preembedding on detection rate of PCa and clinically significant PCa (Gleason score ≥ 7 or > 2 biopsy cores with a Gleason score 6) was compared to 2D GS TRUS-guidance using regression models.

**Results:**

Detection rate of PCa and clinically significant PCa was 39.0 and 24.9% in the 3D GS TRUS cohort compared to 33.5 and 19.0% in the 2D GS TRUS cohort, respectively. On multivariate regression analysis the use of 3D GS TRUS-guidance with preembedding was associated with a significant increase in detection rate of PCa (aOR = 1.33; 95% CI: 1.03–1.72) and clinically significant PCa (aOR = 1.47; 95% CI: 1.09–1.98).

**Conclusion:**

Our results suggest that 3D GS TRUS-guidance with biopsy core preembedding improves PCa and clinically significant PCa detection compared to 2D GS TRUS-guidance. Additional studies are needed to justify the application of these systems in clinical practice.

**Electronic supplementary material:**

The online version of this article (10.1186/s12894-019-0455-7) contains supplementary material, which is available to authorized users.

## Background

Prostate cancer (PCa) is the most common malignancy among Western males [1]. Early detection of PCa reduces PCa-related mortality and reduces the risk of being diagnosed and developing advanced or metastatic disease [2].

PCa detection with systematic prostate biopsies under ultrasound guidance, in men identified with elevated serum prostate-specific antigen (PSA) and / or abnormal digital rectal examination (DRE), is considered as the preferred diagnostic method by guidelines from leading organizations [3, 4]. Although standard two dimensional (2D) greyscale (GS) transrectal ultrasound (TRUS)-guided biopsy is able to detect PCa, it has well known limitations. 2D GS TRUS has low sensitivity and specificity for detection of PCa and is therefore used to guide prostate biopsies rather than used as a diagnostic modality. Even in TRUS-guided biopsy there is still a 20–30% risk of sampling error with underdiagnosis of PCa that requires definitive treatment as a result [5, 6]. There is also poor correlation with final pathology [7–9]. Unfortunately, imaging is not the only part with influence on PCa detection as inadequate tissue packaging in biopsy processing also tends to reduce PCa detection outcomes [10].

To overcome these limitations and improve PCa detection, there is an ongoing focus on new imaging and pathology techniques.

3D GS TRUS is an imaging method that allows simultaneous imaging of the prostate in axial and sagittal plane, followed by a computer reconstructed third coronal plane as well as a 3D image [5]. Effectiveness of 3D GS TRUS in detection and local staging of PCa is controversial as limited studies reported different outcomes [5, 11–13]. However, the feasibility of 3D TRUS in assisting biopsy needle guidance was recognised in phantom and patient studies [14–16]. More recently, Peltier et al. reported significant higher detection rates of clinically significant PCa using a 3D TRUS-guided biopsy system in clinical practice [17].

Biopsy processing with preembedding methods of prostate biopsy cores result in optimal tissue presentation with preservation of shape and avoidance of artefacts and deformation, and have shown to improve histologic yield [18, 19]. Moreover, Rogatsch et al. demonstrated that the improvement of histologic yield using an optimized preembedding method led to a higher frequency of PCa diagnosis [20].

Based on these findings, prostate biopsy sessions in our institution are performed since September 2013 with a phantom validated 3D GS TRUS-guided biopsy system (Navigo™ workstation, UC-care Medical Systems Israel) and a semi-automated prostate biopsy core preembedding method proven to improve histologic yield (the SmartBX™ system, UC-care Medical Systems Israel) [15, 21]. The purpose of the present study was to investigate the value of 3D GS TRUS-guidance and biopsy core preembedding in the detection of (clinically significant) PCa compared to conventional 2D GS TRUS-guidance in a consecutive cohort of men undergoing systematic prostate biopsies due to a clinical suspicion of PCa.

## Methods

In this retrospective cohort study, all consecutive men who underwent prostate biopsies between January 2007 and January 2016 in the Urology Department at the Jeroen Bosch Hospital in ‘s Hertogenbosch, the Netherlands, were included. Patient files were consulted for relevant information. Prostate biopsy sessions performed in patients with no prior history of PCa and a serum PSA level between 4.0 and 10.0 μg/L or PSA < 4.0 μg/L and a suspicious DRE were included in the study. We divided the eligible prostate biopsy sessions into two cohorts. From January 2007 up to September 2013 consecutive biopsy sessions were performed in the 2D TRUS cohort. The 3D TRUS cohort consisted of the consecutive biopsy sessions with preembedding from September 2013 up to January 2016.

### 2D TRUS cohort protocol

All prostate biopsy sessions were performed with prophylactic antibiotics in the lateral decubitus position. The prostate gland was assessed and scanned and prostate volume measurements were done using the ellipsoid formula, using a BK medical ultrasound machine (type 2202) and BK medical sidefire probe (type 8808, 6–10 MHz). A 12-core biopsy protocol, under periprostatic local anaesthesia, was then performed consisting of biopsies on both sides in the peripheral zone, 2 biopsies of each base, mid and apex. The biopsy was performed using a spring-loaded gun with 18-gauge needles (Bard Magnum biopsy gun). Biopsy cores were placed in two vials, each vial containing 6 cores of one prostate lobe. Prostate cores were processed and examined in the hospital pathology laboratory. Number of cores per vial, length of biopsy cores, number of positive biopsy cores, ISUP 2005 Gleason score and tumour volume% of biopsy cores (since 2012) were reported.

### 3D TRUS cohort protocol

**Fig. 1:**
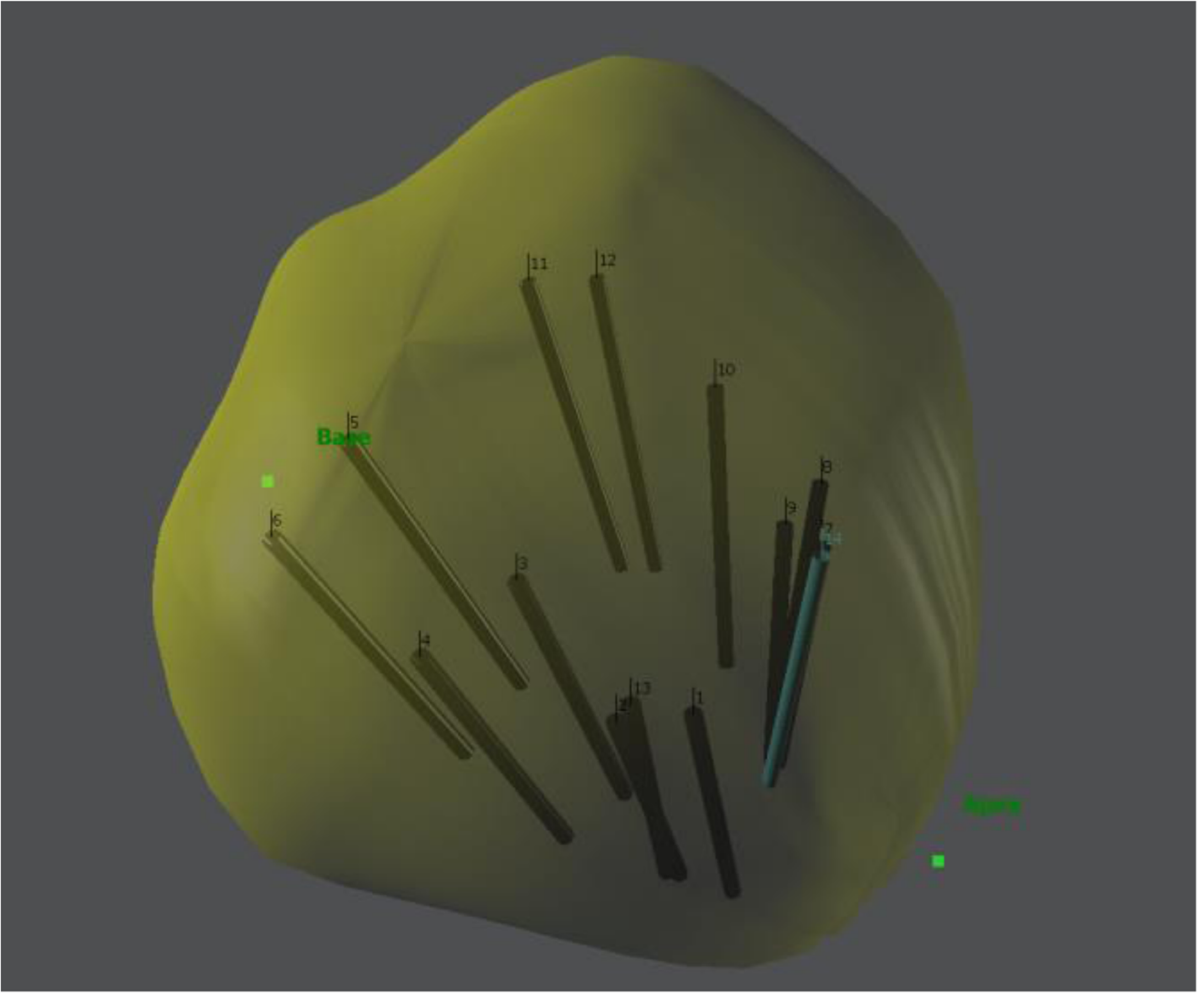
Navigo™ 3D model with biopsy locations

The 3D GS TRUS protocol is basically similar to the 2D TRUS protocol. The 3D GS TRUS system (Navigo™ workstation) was incorporated side by side in the room with 2D ultrasound images transferred and displayed on the 3D TRUS screen. A 3D model of the prostate was built after prostate volume measurements and planimetry was done. The outline of the prostate border was manually traced and recorded on a slice-by-slice basis. In real time using an electromagnetic system (Fig. 1), the 12-core biopsy protocol was performed with tracking, displaying and recording of biopsy needle trajectory locations. Previously marked regions of interest were shown with colour indications on screen and sampled. Biopsy cores were fixed separately on six different cassettes (two cores of apex, mid and base on both sides) using the Smart-Bx device (Additional file 2). SmartBx™ device is a semi-automated prostate biopsy core preembedding method, with a designed membrane to which the cores stay attached throughout pathology lab processing, and placed in two formalin-filled vials (3 cassettes in each vial with two cores of apex, mid and base of one prostate lobe).

In both cohorts, biopsy sessions were performed by different operators. Operator was recorded for analysis and split into experienced (> 100 procedures/year and > 10 years of experience using TRUS guided biopsies) and less experienced (< 100 procedures/year). The following primary outcomes were compared: detection rate of PCa and detection rate of clinically significant (Gleason score ≥ 7 or Gleason score 6 with at least 3 positive biopsy cores). The following secondary outcomes were compared in patients with PCa at biopsy: amount of positive biopsy cores; ratio positive biopsy cores per total biopsy cores; tumour volume% of biopsy cores and Gleason score.

### Statistical

Statistical analyses were performed using SPSS Statistics 23.0®. Descriptive statistics were used to summarize baseline characteristics. Continuous variables were presented with means and standard deviations (SD) or median and interquartile ranges (IQR) based on symmetrical distribution with the independent two sample t-test or Mann-Whitney U test used to assess differences, respectively. Percentage scores were presented for categorical variables with differences assessed using the Pearson Chi-Square test. To determine association between (clinically significant) PCa and the 3D GS TRUS-guided system with preembedding we calculated the crude and adjusted odds ratio with their 95% confidence intervals (CI) for each outcome compared to the 2D GS TRUS cohort using univariate and multivariate logistic regression analyses. Covariables were entered on multivariate analysis, selected based on literature and/or performance: inclusion in the adjusted model when addition of the variable resulted in at least 10% change in the regression coefficient. A *P* <  0.05 was considered statistically significant in all analyses [3, 22–24].

## Results

**Fig. 2:**
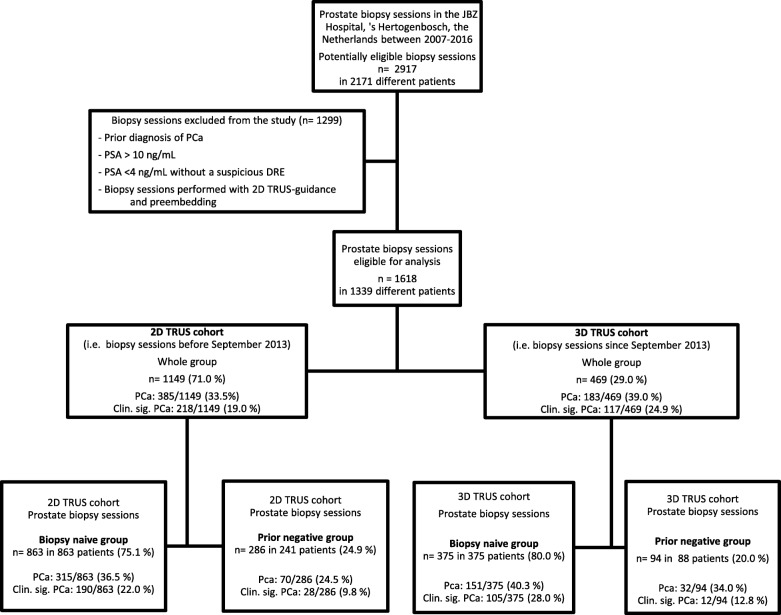
Study population

Between January 2007 and January 2016 we performed 2917 prostate biopsy sessions in 2171 different patients (Fig. 2). 2D TRUS biopsies were performed from 2007 to 2013 and 3D TRUS biopsies were performed from 2013 to 2016. A total of 1618 prostate biopsy sessions in 1339 different patients met inclusion criteria. 469 (29.0%) and 1149 (71.0%) biopsy sessions were performed in the 3D TRUS and 2D TRUS cohort respectively. Patients with missing data on clinical, biopsy and pathology variables (PSA *n* = 4, DRE *n* = 112, prostate volume *n* = 13, length of cores *n* = 20) were excluded for multivariate analyses. Additionally, information on primary outcome was inadequate for 45 biopsy sessions and therefore these sessions were revised by one uropathologist.

### Patient and biopsy characteristics

Several characteristics differed among the two cohorts for whole group and subgroups (biopsy naive and prior negative biopsy sessions only) analyses (Table 1). For whole group analysis, prostate biopsy sessions in the 3D TRUS cohort were more likely done in older patients (mean age in years; 64.6 vs. 63.0, *p* <  0.001), in patients with larger prostate volumes (median volume in cc; 46 vs. 40, *p* <  0.001), in a biopsy naive setting (80.0% vs. 75.1%, *p* <  0.05) and by the experienced operator (73.6% vs. 43.3%, *p* <  0.001) compared to the 2D TRUS cohort. The amount of biopsy cores taken per session was comparable in both cohorts (mean cores 12.3 vs. 12.3, *p* = 0.555), while fragmentation of biopsy cores occurred significantly less often in the 3D TRUS cohort (37.7% vs. 1.9%, *p* <  0.001). The length of biopsy cores in millimetres was significantly larger in the 3D TRUS cohort (mean length in mm; 16.08 vs. 12.38, *p* <  0.001).

**Table 1:** Patient and biopsy characteristics

	Total	2D TRUS	3D TRUS	*P* value
Whole group (1339 patients**)**				
Number of biopsy sessions	*N* = 1618	N = 1149 (71.0%)	*N* = 469 (29.0%)	
Age in years, mean (SD)	63.4 (SD: 6.30)	63.0 (SD: 6.27)	64.6 (SD: 6.24)	< 0.001
PSA in ng/mL, mean (SD)	6.92 (SD: 1.84)	6.95 (SD: 1.85)	6.84 (SD: 1.84)	0.248
DRE:				
• benign, n (%)	1028 (68.3%)	739 (69.3%)	289 (65.7%)	0.167
• suspicious, n (%)	478 (31.7%)	327 (30.7%)	151 (34.3%)	
Prostate volume in ml, median (IQR)	41 (30–56)	40 (30–54)	46 (33–63)	< 0.001
Type of biopsy sessions:				
• Biopsy naive, n (%)	1238 (76.5%)	863 (75.1%)	375 (80.0%)	0.037
• Prior negative, n (%)	380 (23.5%)	286 (24.9%)	94 (20.0%)	
Type of operator:				
• Experienced, n (%)	843 (52.1%)	498 (43.3%)	345 (73.6%)	< 0.001
• Less experienced, n (%)	775 (47.9%)	651 (56.7%)	124 (26.4%)	
Biopsy cores per session, mean (SD)	12.3 (SD 1.04)	12.3 (SD 1.05)	12.3 (SD 1.02)	0.555
Biopsy sessions with fragmentation, n (%)	442 (27.3%)	433 (37.7%)	9 (1.9%)	< 0.001
Length of biopsy cores in mm, mean (SD)^a^	13.84 (SD: 2.98)	12.38 (SD: 2.82)	16.08 (SD: 1.41)	< 0.001
Biopsy naïve group (1238 patients)				
Number of biopsy sessions	*N* = 1238	*N* = 863 (69.7%)	*N* = 375 (30.3%)	
Age in years, mean (SD)	63.5 (SD: 6.46)	63.0 (SD: 6.43)	64.7 (SD: 6.35)	< 0.001
PSA in ng/mL, mean (SD)	6.73 (SD: 1.87)	6.72 (SD: 1.87)	6.74 (SD: 1.86)	0.877
DRE:				
• benign, n (%)	777 (66.9%)	556 (68.6%)	221 (63.0%)	0.063
• suspicious, n (%)	385 (33.1%)	255 (31.4%)	130 (37.0%)	
Prostate volume in ml, median (IQR)	40 (30–55)	40 (29–52)	44 (32–60)	< 0.001
Type of operator:				
• Experienced, n (%)	643 (51.9%)	368 (42.6%)	275 (73.3%)	< 0.001
• Less experienced, n (%)	595 (48.1%)	495 (57.4%)	100 (26.7%)	
Biopsy cores per session, mean (SD)	12.2 (SD 0.79)	12.2 (SD 0.80)	12.2 (SD 0.77)	0.779
Biopsy sessions with fragmentation, n (%)	334 (27.0%)	325 (37.7%)	9 (2.4%)	< 0.001
Length of biopsy cores in mm, mean (SD)^a^	13.84 (SD: 2.96)	12.33 (SD: 2.78)	16.07 (SD: 1.40)	< 0.001
Prior negative group (321 patients)				
Number of biopsy sessions	*N* = 380	*N* = 286 (75.3%)	*N* = 94 (24.7%)	
Prior negative sessions specified:				
• Second session	300 (78.9%)	225 (78.7%)	75 (79.8%)	0.658
• Third session	64 (16.8%)	47 (16.4%)	17 (18.1%)	
• Fourth session	14 (3.7%)	12 (4.2%)	2 (2.1%)	
• Fifth session	2 (0.5%)	2 (0.7%)	0	
Age in years, mean (SD)	63.1 (SD: 5.79)	62.8 (SD: 5.80)	63.9 (SD: 5.73)	0.135
PSA in ng/mL, mean (SD)	7.54 (SD: 1.60)	7.65 (SD: 1.54)	7.20 (SD: 1.73)	0.023
DRE:				
• benign, n (%)	251 (73.0%)	183 (71.8%)	68 (76.4%)	0.396
• suspicious, n (%)	93 (27.0%)	72 (28.2%)	21 (23.6%)	
Prostate volume in ml, median (IQR)	46 (32–62)	44 (31–58)	54 (39–70)	< 0.001
Type of operator:				
• Experienced, n (%)	200 (52.6%)	130 (45.5%)	70 (74.5%)	< 0.001
• Less experienced, n (%)	180 (47.4%)	156 (54.5%)	24 (25.5%)	
Biopsy cores per session, mean (SD)	12.6 (SD 1.56)	12.7 (SD 1.53)	12.6 (SD 1.67)	0.519
Biopsy sessions with fragmentation, n (%)	113 (29.7%)	113 (39.2%)	0	< 0.001
Length of biopsy cores in mm, mean (SD)^a^	13.81 (SD: 3.05)	12.54 (SD: 2.96)	16.10 (SD: 1.48)	< 0.001

^a^Biopsy sessions with fragmentation were excluded for these analyses

### Detection rate

In the 3D TRUS cohort, a total of 183 out of 469 (39.0%) and 117 out of 469 (24.9%) were found positive for PCa and clinically significant PCa compared to 335 out of 1149 (33.5%) and 218 out of 1149 (19.0%) in the 2D TRUS cohort, respectively (Table 2). On univariate analysis the 3D TRUS cohort was significantly associated with detection of PCa (OR = 1.27, 95% CI 1.02–1.59) and clinically significant PCa (OR 1.42, 95% CI 1.10–1.83). On multivariate regression analysis with age, DRE, prostate volume and type of operator included as covariates, the 3D TRUS cohort remained significantly associated with detection of PCa (aOR 1.33, 95% CI 1.03–1.72) and clinically significant PCa (aOR 1.47, 95% CI 1.09–1.98). The association between 3D TRUS and detection of (clinically significant) PCa on multivariate regression analysis for the subgroups demonstrated significance for clinically significant PCa in the biopsy naive group (aOR 1.41, 95% CI 1.02–1.96) and for PCa in the prior negative group (aOR 1.78, 95% CI (1.04–3.04) (Table 2).

**Table 2:** Detection rate of all prostate cancer and clinically significant prostate cancer

Whole group			
*Detection rate of PCa*			
Biopsy cohort:	n/N (%)	Crude OR (95% CI)	aOR^a^ (95% CI)
Total	568/1618 (35.1%)		
3D TRUS	183/469 (39.0%)	1.27 (1.02–1.59)	1.33 (1.03–1.72)
2D TRUS	385/1149 (33.5%)	Reference	Reference
*Detection rate of clinically significant PCa*			
	n/N (%)	Crude OR (95% CI)	aOR^a^ (95% CI)
Total	335/1618 (20.7%)		
3D TRUS	117/469 (24.9%)	1.42 (1.10–1.83)	1.47 (1.09–1.98)
2D TRUS	218/1149 (19.0%)	Reference	Reference
Biopsy naive group			
*Detection rate of PCa*			
	n/N (%)	Crude OR (95% CI)	aOR^b^ (95% CI)
Total	466/1238 (37.6%)		
3D TRUS	151/375 (40.3%)	1.17 (0.92–1.50)	1.20 (0.90–1.60)
2D TRUS	315/863 (36.5%)	Reference	Reference
*Detection rate of clinically significant PCa*			
	n/N (%)	Crude OR (95% CI)	aOR^b^ (95% CI)
Total	295/1238 (23.8%)		
3D TRUS	105/375 (28.0%)	1.38 (1.04–1.82)	1.41 (1.02–1.96)
2D TRUS	190/863 (22.0%)	Reference	Reference
Prior negative group			
*Detection rate of PCa*			
	n/N (%)	Crude OR (95% CI)	aOR^c^ (95% CI)
Total	102/380 (26.8%)		
3D TRUS	32/94 (34.0%)	1.59 (0.96–2.64)	1.78 (1.04–3.04)
2D TRUS	70/286 (24.5%)	Reference	Reference
*Detection rate of clinically significant PCa*			
	n/N (%)	Crude OR (95% CI)	aOR^d^ (95% CI)
Total	40/380 (10.5%)		
3D TRUS	12/94 (12.8%)	1.35 (0.66–2.77)	–
2D TRUS	28/286 (9.8%)	Reference	Reference

^a^adjusted Odds Ratio: adjusted for age, DRE, prostate volume and physician (PSA and Type of biopsy session resulted in < 10% change in β)

^b^ adjusted Odds Ratio: adjusted for age, DRE and prostate volume and physician (PSA resulted in < 10% change in the β)

^c^ adjusted Odds Ratio: adjusted for age and Log (prostate volume), (age, PSA, DRE and physician resulted in < 10% change in the β)

^d^ the number of events per variable are too low to be analyzed in multivariable logistic regression analysis

### PCa positive biopsies: biopsy characteristics and Gleason score

Tumour biopsy characteristics between the 3D GS TRUS cohort and the 2D GS TRUS cohort in patients with positive PCa biopsies were not statistically different as the amount of positive biopsy cores, the ratio positive biopsy cores out of the total biopsy cores and the percentage of tumour volume per PCa biopsy session did not differ between both groups (Additional file 3). The biopsy Gleason score among patients with PCa positive biopsies was significantly different between the 3D TRUS and 2D TRUS cohort in the whole group and biopsy naive subgroup analyses as more clinically significant and Gleason score ≥ 7 PCa was found in the 3D TRUS cohort (*P* <  0.05) (Additional file 4).

## Discussion

Limited studies with small sample sizes have been performed to assess the value of 3D GS TRUS in guiding prostate biopsies. Peltier et al. published the first cohort study into 3D TRUS-guided prostate biopsy and detection rate of PCa. In 220 consecutive biopsy naive men, detection rate of PCa was significantly higher in the 3D TRUS group using the Urostation (Koelis®) compared with 2D TRUS (50.0% versus 33.6%, *p* < 0.05) [17]. In contrary, a previous study carried out in our institution with 325 men showed no added value of 3D TRUS guidance compared to 2D with all biopsy cores preembedded (aOR 0.79, 95% CI 0.47–1.34) [25].

This present study, covering a period of 9 full years with 1618 prostate biopsy sessions, demonstrated higher detection rates of PCa and clinically significant PCa with the use of 3D TRUS-guidance and preembedding compared to conventional 2D GS TRUS-guidance. These findings may be explained by different hypotheses. On one side, 3D TRUS could have improved detection as it offers the ability to accurately register the location of the biopsy needle tract within the gland, possibly leading to better distribution of biopsy cores; instead needle placement with 2D-guidance is not always homogeneously distributed and tends to cluster.[15, 26–28] Besides, length of biopsy cores was significantly larger and fragmentation occurred significantly less in the 3D GS TRUS cohort possibly also aiding detection of PCa as Iczkowski et al. and Rogatsch et al. demonstrated in their studies that length of biopsy tissue significantly correlates with PCa detection rate and higher frequency of PCa diagnosis was found with an improved preembedding method, respectively [19, 20, 25].

Multiparametric MRI (mpMRI) of the prostate is increasingly used in the diagnostic pathway of PCa and three large studies, evaluating the detection rates of an MRI-targeted biopsy approach and TRUS guided systematic biopsy approach, have recently been performed in biopsy-naïve men [30–32].

While the PRECISION trial demonstrated that an MRI targeted biopsy approach detected significantly more clinically significant PCa in comparison with a TRUS systematic biopsy approach both the MRI First and 4 M Study demonstrated comparable detection rates of clinically significant PCa between the standard TRUS systematic biopsy and MRI targeted biopsy approach.

Obtaining an mpMRI before biopsy improves the detection of clinically significant PCa but at present does not avoid the need for systematic biopsy as shown in the systematic review from Moldovan et al. and the MRI FIRST study where 10 to 15% of clinically significant PCa were still missed in men with a negative mpMRI [31, 33]. Even in the Dutch 4 M study with high-quality MRI standards, 7% (21/317) of all men with a suspicious mpMRI scan had clinically significant PCa only on systematic biopsy [32]. A high-quality TRUS systematic biopsy, possibly with the use of 3D TRUS-guidance and preembedding, could therefore still be important in the current diagnostic setting where mpMRI is also included.

On the other side, as a retrospective study, our analysis has important limitations that could have influenced the outcome. First of all, biopsy sessions after September 2013 were performed with 3D TRUS guidance and the preembedding method; as a consequence, their independent influence on the detection rate of PCa cannot be assessed. However as both procedures act on different aspects of PCa diagnosis, a joint positive effect was hypothesized over masking of independent results. Secondly, difference in study time period could have had an impact on PCa detection rate as different unidentified factors influencing PCa detection rate could have changed during these time periods. Prostate biopsy selection in both cohorts however was not based on important pre-biopsy tests such as mpMRI and/or novel biomarkers. Both cohorts differed in baseline characteristics with older age, lower prostate volume and a biopsy naive setting all associated with higher odds of PCa and significant PCa on univariate and multivariate analysis in our study (Additional file 1: Table S1). Beside this, we decided in our study to include the Large Grade Group 1 (GS 3 + 3) as clinically significant PCa. Recent EAU guidelines use the definition of GG ≥ 2 (GS ≥3 + 4) for clinical significant PCa. This matches with the newly introduced ISUP scoring system, where no separation is made between large and small GG 1 (GS 3 + 3) PCa [29]. We decided to include large GS 3 + 3 = 6 PCa as clinically significant as this is currently still used as a criteria for active surveillance versus radical treatment in low-risk PCa patients.

In addition, experience level of operators was various. Although a correction strategy was implemented, residual confounding could still be possible. Last, 32.4% (944/2917) of all our prostate biopsy sessions were excluded on the basis of PSA inclusion criteria. In line with the literature we excluded men with high PSA levels as improved detection of significant PCa cancer is most necessary in the PSA grayzone from 4 to 10 ng/mL, while men with higher PSA levels tend to be diagnosed accurately with TRUS-guided systematic biopsies [30–32].

As a result, association between 3D TRUS-guidance with preembedding and detection of PCa could be partially biased on the basis of confounding and selection.

Despite these limitations in relation to the detection of PCa, there are other possible clinical applications for our approach. The 3D TRUS system is not only developed for direct imaging and navigation, but also for procedure planning with recording and integration of exact locations of biopsy cores and updating of pathology results. With the possibility to assess the exact spatial coordinates of each previous needle trajectory, a ‘biopsy map’ can be built based on (positive) previous core taken and adequate sample sites can potentially be chosen in prior negative patients or in patients in active surveillance. Additionally, in our study biopsy core length and Gleason score were significantly higher in the cohort with 3D GS TRUS-guidance and preembedding. As more biopsy core length from a presumably spatial better biopsied prostate gland can be pathologically examined, correlation with prostatectomy specimens could possibly be improved and thereby prevent men with unfavourable disease to be placed in active surveillance or less aggressive treatments. However caution should be exercised for interpretation of Gleason score differences between both cohorts as Gleason grading of PCa was updated at the end of 2014 during an ISUP Consensus Conference [29].

As a consequence, higher Gleason scores in the 3D TRUS cohort could be partially the result of these new grading pattern definitions. We will therefore investigate these potential applications in future prospective studies and pathology concordance studies. These findings highlight the need for future research regarding the complementary value of 3D TRUS guidance and preembedding in the combination with mpMRI and other new diagnostic applications.

## Conclusion

The current study suggests an added value of 3D TRUS-guidance and preembedding compared to conventional 2D GS TRUS-guidance regarding detection rate of PCa and clinically significant PCa among patients undergoing prostate biopsies. At the same time, 3D TRUS-guidance and preembedding provided significant more biopsy core tissue length and higher biopsy Gleason scores. Additional prospective studies and studies concerning pathology correlation are needed to justify the routine application of 3D GS TRUS-guidance and biopsy core preembedding.

## Additional files

Additional file 1: **Table S1.** Univariate and multivariate logistic regression analyses of individual risk factors for detection of prostate cancer and clinically significant prostate cancer. (DOCX 14 kb)

Additional file 2: SmartBx^TM^ device prostate biopsy core preembedding. (PPTX 95 kb)

Additional file 3: Tumour biopsy characteristics among patients with positive biopsies. (XLSX 12 kb)

Additional file 4: Biopsy Gleason score among patients with positive biopsies. (XLSX 13 kb)

## Abbreviations

2D: Two-dimensional
3D: Three-dimensional
CI: Confidence interval
DRE: Digital rectal examination
GS: Greyscale
IQR: Interquartile range
mpMRI: Multiparametric Magnetic Resonance Imaging
OR: Odds ratio
PCa: Prostate cancer
PSA: prostate-specific antigen
SD: Standard deviation
TRUS: Transrectal ultrasound
