# Supplementary material for: Three-dimensional greyscale transrectal ultrasound-guidance and biopsy core preembedding for detection of prostate cancer: Dutch clinical cohort study
Source: BMC Urol. 2019 Apr 16;19:23. doi: 10.1186/s12894-019-0455-7 (PMC6469087; doi:10.1186/s12894-019-0455-7)
Supplement: Supplementary file 1 — Table S1. Univariate and multivariate logistic regression analyses of individual risk factors for detection of prostate cancer and clinically significant prostate cancer. (DOCX 14 kb) [file 12894_2019_455_MOESM1_ESM.docx]

**Supporting Table 1. Univariate and multivariate logistic regression analyses of individual risk factors for detection of prostate cancer and clinically significant prostate cancer**

| Whole group | | | | | | |
| --- | --- | --- | --- | --- | --- | --- |
|  | Univariate | | | Multivariate | | |
| Risk factor | OR | 95% CI | P | OR | 95 % CI | P |
| *Prostate cancer* | | | | | | |
| Age (years) | 1.05 | 1.03-1.07 | <0.001 | 1.06 | 1.04-1.08 | <0.001 |
| PSA (ng/mL) | 1.02 | 0.97-1.08 | 0.490 | 1.09 | 1.02-1.16 | 0.008 |
| DRE | 2.05 | 1.64-2.56 | <0.001 | 1.77 | 1.39-2.25 | <0.001 |
| Prostate volume (cc) | 0.97 | 0.97-0.98 | <0.001 | 0.97 | 0.97-0.98 | <0.001 |
| Type of biopsy session* | 1.65 | 1.28-2.12 | <0.001 | 1.47 | 1.11-1.96 | 0.007 |
| Type of operator† | 1.24 | 1.01-1.53 | 0.037 | 1.08 | 0.86-1.37 | 0.503 |
| Biopsy technique‡ | 1.27 | 1.02-1.59 | 0.035 | 1.36 | 1.05 | 0.018 |
| *Significant prostate cancer* | | | | | | |
| Age (years) | 1.07 | 1.05-1.09 | <0.001 | 1.08 | 1.05-1.10 | <0.001 |
| PSA (ng/mL) | 1.04 | 0.98-1.11 | 0.221 | 1.14 | 1.06-1.23 | <0.001 |
| DRE | 2.80 | 2.17-3.61 | <0.001 | 2.38 | 1.80-3.15 | <0.001 |
| Prostate volume (cc) | 0.97 | 0.96-0.97 | <0.001 | 0.96 | 0.95-0.97 | <0.001 |
| Type of biopsy session* | 2.66 | 1.87-3.78 | <0.001 | 2.58 | 1.74-3.83 | <0.001 |
| Type of operator† | 1.39 | 1.09-1.77 | 0.009 | 1.21 | 0.91-1.61 | 0.184 |
| Biopsy technique‡ | 1.42 | 1.10-1.83 | 0.007 | 1.47 | 1.08-1.99 | 0.014 |

* Type of biopsy session: prior negative as reference. † Type of operator: less experienced as reference. ‡ Biopsy technique: 2D TRUS as reference
