# Supplementary figures and images for: Three-dimensional greyscale transrectal ultrasound-guidance and biopsy core preembedding for detection of prostate cancer: Dutch clinical cohort study
Source: BMC Urol. 2019 Apr 16;19:23. doi: 10.1186/s12894-019-0455-7 (PMC6469087; doi:10.1186/s12894-019-0455-7)

## Slide 1
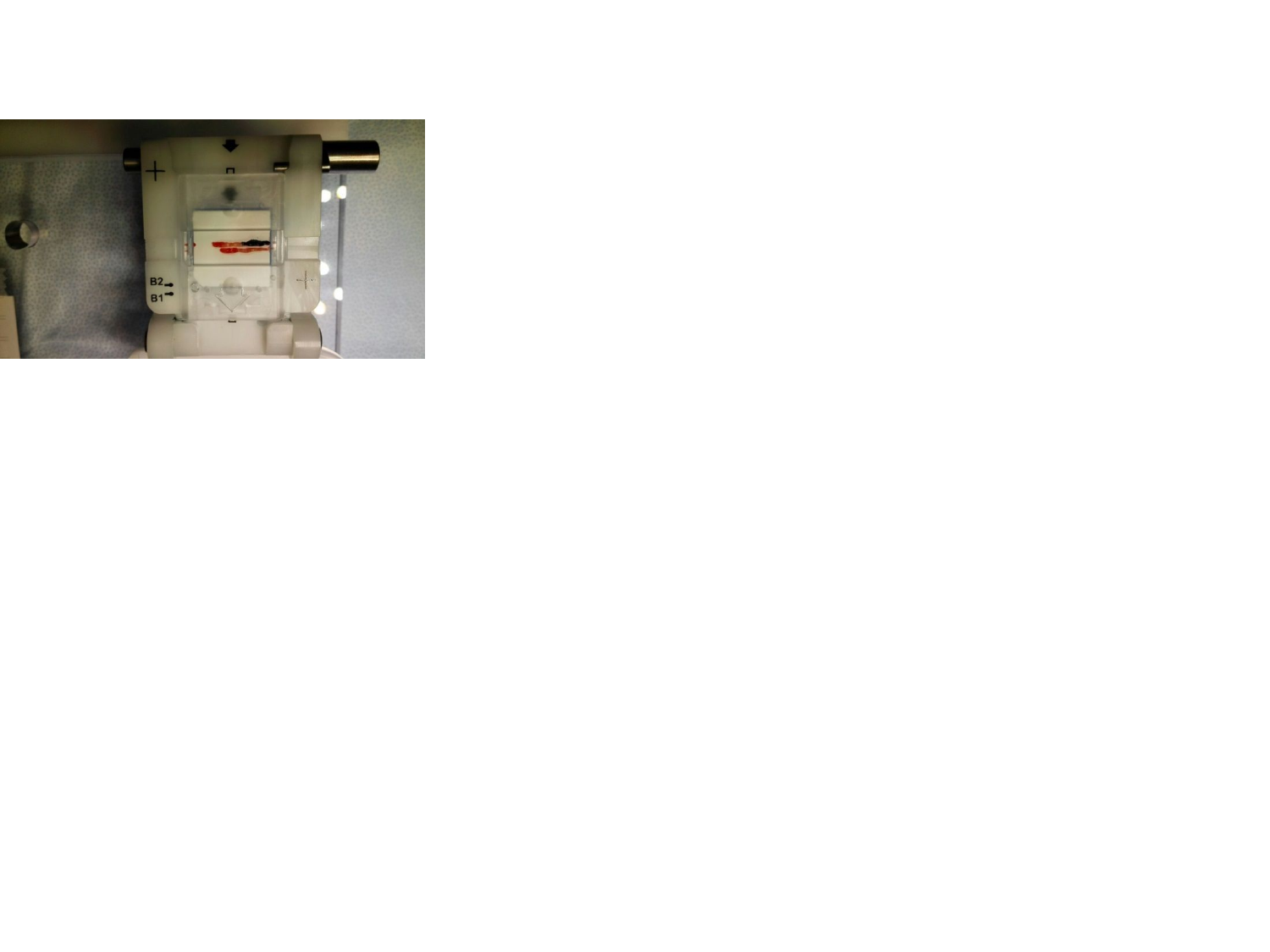

Supplement: Supplementary file 2 — SmartBxTM device prostate biopsy core preembedding. (PPTX 95 kb) [file 12894_2019_455_MOESM2_ESM.pptx]
